# Advanced oxidation protein products induce microglia-mediated neuroinflammation via MAPKs-NF-κB signaling pathway and pyroptosis after secondary spinal cord injury

**DOI:** 10.1186/s12974-020-01751-2

**Published:** 2020-03-20

**Authors:** Zhongyuan Liu, Xinqiang Yao, Wangsheng Jiang, Wei Li, Siyuan Zhu, Congrui Liao, Lin Zou, Ruoting Ding, Jianting Chen

**Affiliations:** Department of Spinal Surgery, Nanfang Hospital, Southern Medical University, Guangzhou, 510515 Guangdong China

**Keywords:** Advanced oxidation protein products, Inflammatory response, Spinal cord injury, ROS-dependent, MAPKs-NF-κB signaling pathway, NLRP3-mediated pyroptosis

## Abstract

**Background:**

Inflammatory response mediated by oxidative stress is considered as an important pathogenesis of spinal cord injury (SCI). Advanced oxidation protein products (AOPPs) are novel markers of oxidative stress and their role in inflammatory response after SCI remained unclear. This study aimed to investigate the role of AOPPs in SCI pathogenesis and explore the possible underlying mechanisms.

**Methods:**

A C5 hemi-contusion injury was induced in Sprague-Dawley rats to confirm the involvement of AOPPs after SCI. For in vivo study, apocynin, the NADPH oxidase inhibitor was used to study the neuroprotective effects after SCI. For in vitro study, the BV2 microglia cell lines were pretreated with or without the inhibitor or transfected with or without small interference RNA (siRNA) and then stimulated with AOPPs. A combination of molecular and histological methods was used to clarify the mechanism and explore the signaling pathway both in vivo and in vitro. One-way analysis of variance (ANOVA) was conducted with Bonferroni post hoc tests to examine the differences between groups.

**Results:**

The levels of AOPPs in plasma and cerebrospinal fluid as well as the contents in the spinal cord showed significant increase after SCI. Meanwhile, apocynin ameliorated tissue damage in the spinal cord after SCI, improving the functional recovery. Immunofluorescence staining and western blot analysis showed activation of microglia after SCI, which was in turn inhibited by apocynin. Pretreated BV2 cells with AOPPs triggered excessive generation of reactive oxygen species (ROS) by activating NADPH oxidase. Increased ROS induced p38 MAPK and JNK phosphorylation, subsequently triggering nuclear translocation of NF-κB p65 to express pro-inflammatory cytokines. Also, treatment of BV2 cells with AOPPs induced NLRP3 inflammasome activation and cleavage of Gasdermin-d (GSDMD), causing pyroptosis. This was confirmed by cleavage of caspase-1, production of downstream mature interleukin (IL)-1β and IL-18 as well as rupture of rapid cell membrane.

**Conclusions:**

Collectively, these data indicated AOPPs as biomarkers of oxidative stress, modulating inflammatory response in SCI by multiple signaling pathways, which also included the induction of NADPH oxidase dependent ROS, and NLRP3-mediated pyroptosis, and activation of MAPKs and NF-κB.

## Introduction

Traumatic spinal cord injury (SCI) is a devastating injury that often occurs as a result of motor and neurological dysfunction [1]. Every year, more than 17,000 new cases of spinal cord injury (SCI) are reported in the USA, contributing to over 282,000 people with motor and neurological dysfunctions [2]. Secondary injury involves microglial activation and neuroinflammation, releasing reactive oxygen species (ROS) and pro-inflammatory cytokines [3].

Neuroinflammation plays a key role in the secondary injury of SCI. Microglia are major innate immune cells of the central nervous system (CNS) and play a prominent role in neuroinflammation [4]. Microglia are rapidly activated in response to SCI, resulting in the upregulation of pro-inflammatory cytokines mediated by multiple signaling cascades. While excessive microglial activation can cause overproduction of pro-inflammatory factors (such as IL-1β, TNF-α) and ROS, which might in turn amplify inflammation and aggravate secondary injury [5, 6].

Mitogen-activated protein kinases (MAPKs) family, including extracellular signal-regulated kinase (ERK), p38 MAPK, and c-Jun N-terminal kinase, are a group of signaling molecules that play an important role in the expression of pro-inflammatory cytokines. ROS mediate MAPK signaling pathway, inducing inflammatory response. The activation of MAPK signaling pathway contributed to the mediation of pro-inflammatory factors by microglia after acute SCI. Previous study has demonstrated that hyper-phosphorylation of MAPK molecules finally activated the transcription factor NF-κB and the follow-up inflammatory reactions. Moreover, neuroinflammation following SCI can be induced by activated microglia through nuclear factor-κB (NF-κB) signaling pathway, which plays an important role in neuroinflammation and immune response [7]. In the spinal cord, direct inhibition of NF-κB signaling pathway prevents microglia from expressing certain pro-inflammatory cytokines, such as IL-6, TNF-α, or IL-1β, which thereby reduces secondary damage caused by inflammation [8].

Recent studies have demonstrated the vital role played by microglia in inflammatory processes through pyroptosis. Stephanie and colleagues have reported that penetrating brain injury induces microglial pyroptosis via inflammasome activation [9, 10]. Pyroptosis is defined as an inflammatory mechanism that regulates cell death, which is mainly mediated by inflammasomes and Gasdermin-d (GSDMD) by releasing pro-inflammatory mediators including IL-1β and IL-18 [11]. Inflammasomes are a group of cytosolic protein complexes in immune cells that plays a critical role in host defense mechanisms and cellular damage. Several types of inflammasomes have been described in CNS diseases, which included NLRP1, NLRP2, NLRP3, NLRC4, and AIM2. Among all these, NLRP3 is so far the most commonly studied and the best characterized inflammasome in the microglia [12, 13]. NLRP3 inflammasome consists of NLRP3, apoptosis-associated speck-like protein containing a caspase recruitment domain (ASC) and caspase-1, and is a multiprotein complex that mediates the activation of caspase-1, which subsequently promotes the maturation and release of IL-1β and IL-18 [14]. Recent research showed that SCI triggers NLRP3 inflammasome activation in the microglia of the spinal cord [15]. At present, three mechanisms have been put forwarded for the activation of NLRP3 inflammasome, including ROS activation, lysosome rupture, and ion channel gating [16]. Previous study showed that ROS mediated by NADPH oxidase (NOX) is considered as one of the most important triggering factors to activate NLRP3 inflammasome [17].

Advanced oxidation protein products (AOPPs) included dityrosine- and cross-linking protein products, which are considered as novel markers of oxidative stress [18]. AOPPs are regarded as biomarkers of oxidative stress in different neuroinflammatory diseases, such as Parkinson’s disease, multiple sclerosis, and amyotrophic disease [19]. Previous studies have shown that AOPPs are not only the products of oxidative stress, but are also the activators of ROS [20]. The role of AOPPs in the activation of NADPH oxidase has been described previously, and is regarded as the major source of ROS generation. However, the mechanism of ROS generation triggered by AOPPs in the pathophysiology of SCI has not yet been studied.

The present study aimed to investigate the role of AOPPs in secondary SCI and their potential molecular mechanisms both in vivo and in vitro. In the present study, the mouse microglial cell line BV2 was used in vitro due to its close resemblance with primary microglia [21]. We are the first to report the increase of AOPPs after SCI in rats. AOPPs induced NADPH-mediated ROS to activate MAPKs-NF-κB signaling pathway and NLRP3-mediated pyroptosis. This study was conducted to provide insights into the mechanisms of AOPPs-induced inflammatory processes after SCI.

## Methods and materials

### Animals

Adult male Sprague-Dawley rats (280–320 g) were used for animal experiments. Rats were housed five per cage and received food and water ad libitum with 12:12 h light/dark cycle. All experiments were approved by the Laboratory Animal Care and Use Committee of Nanfang Hospital, Southern Medical University.

### Surgical procedure

All animals were randomly divided into four experimental groups, with 24 rats per group: control group, Sham group, SCI group, and SCI + apocynin group. The rats in the SCI group were divided into 4 subgroups according to the days (3, 7, 14, and 28 days) after SCI. In our study, the rats in the SCI + apocynin group were intraperitoneally administered with apocynin each day at a dose of 100 mg/kg after SCI. Rats underwent a C5 hemi-contusion as previously described [22]. Briefly, a custom-designed clamp was mounted onto the lateral C4–C6 column after a unilateral C5 laminectomy was performed. The impact was centered over to the left side of the C5 segment and then triggered to deliver a set displacement of 2.2 mm at 500 mm/s. The spinal cord tissues, plasma, and cerebrospinal fluid were obtained on days 3, 7, 14, and 28 after SCI from rats anesthetized by pentobarbital sodium (80 mg/kg, i.p.).

### AOPPs-MSA preparation and determination

AOPPs-MSA was prepared in vitro as described previously [23]. In brief, 20 mg/mL MSA solution was incubated with 40 mmol/L hypochlorous acid (HOCL) in PBS (pH = 7.4) for 30 min at room temperature and dialyzed for 24 h against PBS at 4 °C to remove the free HOCL. All samples were passed through a Detoxi-Gel column to remove the contaminated endotoxins. The endotoxin levels in AOPPs-modified MSA and unmodified MSA were then measured by using a Limulus Amoebocyte Lysate kit and were found to be below 0.025 EU/mL. To determine the content of AOPPs, 200 μl of sample or chloramine-T was placed in 96-well plate, followed by 20 μl of acetic acid. The absorbance was read at 340 nm immediately by using a microplate reader. The content of AOPPs in AOPPs-MSA and unmodified MSA were 50.50 ± 2.80 μmol/g protein and 0.25 ± 0.06 μmol/g protein, respectively.

### Assay of plasma cerebrospinal fluid and spinal cord AOPPs levels

The method for determining the levels of AOPPs in the plasma, cerebrospinal, and spinal cord was identical to that used for determining the content of AOPPs in AOPPs-MSA compound.

### Behavioral assessments

The animals were observed on days 0, 3, 7, 14, and 28 after SCI. All behavioral assessments were scored by three individuals who were blinded to grouping.

#### Grooming test

This test was originally developed by Bertelli and Mira to examine the behavioral functional recovery of rats by constructing a brachial plexus reconstruction model. Animals were placed in a clear plastic cylinder with two mirrors placed at angles for clear visibility of the rat’s head. Cool saline was applied to the animal’s head and back with soft gauze prior to the placement of the animal in the cylinder, and the activities of the animals were recorded with a digital camera for 15 min. The individuals were then analyzed by recording and rating with scores as described by Gensel [24].

#### Cylinder rearing test

Forelimb usage was tested during rearing as described previously [25]. Briefly, the rats were placed in a clear plexi-glass cylinder and recorded for 15 min. Two mirrors were placed at 90° angle behind the cylinder for clear visibility of the forelimbs all the time. In total, 20 rearing events were analyzed frame-by-frame. Forelimb usage was presented as the percentage of ipsilateral or contralateral forelimb usage.

#### Forelimb locomotor scale

Forelimb and hindlimb functions were evaluated in an open-field measuring 2.5 × 3 ft and the rats were observed for 4 min. The forelimb function was evaluated based on the FLS created to describe the common deficits in rats with cervical injury [26]. The FLS was a 17-point scale developed based on the Basso, Beattie, and Bresnahan (BBB) score. It was designed to describe the deficits observed in body-weight support, forelimb range of motion during stepping, and paw position on stepping liftoff and landing.

### Cell culture

The murine BV2 microglial cell line was obtained from Shanghai Cell Research Center (Shanghai, China). The cells were cultured in DMEM (4.5 g/L glucose) containing 10% FBS and 1% penicillin/streptomycin at 37 °C in a 5% CO_2_ atmosphere. When the cells reach to approximately 80% confluence, they were digested with trypsin and passaged for additional experiments.

### Cell viability (MTT) assay

MTT viability assay was performed to determine the cell viability after AOPPs-MSA treatment. BV2 cells were cultured with AOPPs-MSA of different concentrations of 50, 100, 200, 400, or 800 μg/mL for 24 h and then the medium was replaced with control medium containing MTT solution (5 mg/mL) in an amount equal to that of 10% culture volume. After incubation for 4 h, the cultures were removed and then dissolved, resulting in the formation of MTT formazan in DMSO. The absorbance was measured at a wavelength of 490 nm.

### Morphological changes of BV2 cells

The BV2 cells that are cultured in 12-well plates are treated with AOPPs-MSA at 50, 100, 200 μg/mL, and LPS for 2 h. After treatment, the medium was removed, and the cells were fixed with 4% formaldehyde for 15 min at room temperature. The fixed BV2 cells were washed twice with PBS for 10 min, and then the morphology of the cells was observed under a light microscope.

### Intracellular ROS measurement

The level of intracellular ROS was measured by a probe 2′,7′-dichlorofluorescein diacetate (DCFH-DA) as described previously [27]. Briefly, the BV2 cells were stimulated with AOPPs-MSA at increasing concentrations or 200 μg/mL AOPPs-MSA for 24 h. Each sample was incubated in 10 μM DCFH-DA for 30 min in darkness. The excitation and emission wavelengths were read at 488 and 525 nm, respectively. The resulting data were normalized using the control values.

### Tissue preparation

On days 3, 7, 14, and 28 after SCI, the animals were deeply anesthetized with sodium pentobarbital (80 mg/kg, i.p.) and transcardially perfused with 0.1 M of phosphate-buffered saline (PBS) followed by treatment with ice-cold 4% paraformaldehyde. Immediately after perfusion, the spinal cord tissue at the lesion site was dissected, post-fixed overnight, and cryoprotected in graded concentrations of sucrose (12%, 18%, and 24%). A 10 mm segment of the cervical cord including the injury epicenter was sectioned in transverse horizontal plane using a cryostat (Leica) at 10 or 20 μm thickness. The 10 μm sections underwent immunofluorescence and DHE staining and the 20 μm sections underwent H&E staining.

### DHE staining

To assess the oxidative stress levels in the spinal cord, frozen spinal cord sections were incubated with 2 μmol/L fluorescent dye dihydroethidium at 37 °C for 30 min in a humidified chamber and protected from light. The images were then captured by fluorescence microscope (Olympus BX63, Japan).

### Hematoxylin and eosin staining

To observe the damage of spinal cord, frozen spinal cord sections were stained with H&E on day 28 after SCI.

### Pyroptosis assay

#### LDH release assays

To assess the AOPPs-induced cytotoxicity in BV2 cells, the release of lactate dehydrogenase (LDH) was measured by using LDH kit according to the manufacturer’s instructions. BV2 cells were seeded in 96-well plates until the desired confluence was reached. The supernatants were then harvested and analyzed for LDH activity using a microplate reader. Also, the percentage of LDH per infection condition was calculated using the formula: percent LDH release (%) = [(infected cell LDH release − spontaneous LDH release)/(total LDH release − spontaneous LDH release)] × 100.

#### PI uptake assays

To confirm the pore formation in AOPPs-induced cell membrane, PI staining was performed as described previously [28]. The BV2 cells were seeded in 24-well plates to reach a proper cell density. After the treatments, the cells were washed with PBS, and then stained with PI and DAPI for 20 min at 37 °C in dark. The cells with red and blue fluorescence were then imaged under confocal laser scanning microscopy.

### Small interfering RNA transfection

siRNA, against mouse Nox4, NLRP3, ASC, Caspase-1, GSDMD, and negative control, were designed and synthesized by RiboBio (Guangzhou, China). siRNA transfection was conducted according to the manufacturer’s instructions. Briefly, the transfection reagent Lipofectamine 2000 (Invitrogen) and siRNA were mixed together in Opti-MEM. The cell culture medium was then replaced with Opti-MEM for 6 h. The cells were then changed to normal medium and continued culturing for 48 h before following the experiments.

### Western blot analysis

BV2 microglial cells were treated with AOPPs at increasing concentrations (50,100, and 200 μg/mL) or 200 μg/mL AOPPs for 24 h. The spinal cord (1 cm) was then homogenized in ice-cold RIPA buffer with 1 mM PMSF, protease, and phosphatase inhibitors and cleared by centrifugation (12,000 rpm, 4 °C, 10 min). For detecting NF-κB p65 translocation, the nuclei proteins were extracted by using a protein extraction kit (Cytoplasmic and Nuclear Protein Extraction Kit, Keygen Biotech, Jiangsu, China). The protein concentration in the supernatant was measured by BCA protein assay kit. Equivalent amounts of extracted proteins (30 μg) were separated by 10% or 12% SDS–poly acrylamide gel electrophoresis, and then electro-blotted onto the PVDF membranes (Millipore, MA, USA). After being blocked at room temperature in 5% blocking buffer (5% BSA in Tris-buffered saline with 0.1% Tween 20) for 1 h, the membranes were incubated overnight at 4 °C with the following primary antibodies: anti-Nox1 (1:1000), anti-Nox4 (1:1000), anti-CD11b (1:2000), anti-AOPP (1:1000), anti- TNF-α (1:1000), anti-IL-1β (1:1000), anti-p-p38 (1:1000), anti-p38 (1:1000), anti-p-JNK (1:1000), anti- JNK (1:1000), and anti-p-ERK (1:1000), anti- ERK (1:1000), anti-p-Iκb-α (1:1000), anti-Iκb-α (1:1000), anti- p-NF-κB p65 (1:1000), anti- NF-κB p65 (1:1000), anti- NLRP3 (1:1000), anti-ASC (1:1000), anti- Caspase-1 (1:1000), and anti-GSDMD (1:1000). The membranes were washed with TBS-T, and then incubated with secondary antibody. The relative intensity of each band was quantified using Gel-Pro Analyzer (Media Cybernetics, Sarasota, FL, USA). Anti-GAPDH (1:5000) and anti- Histone H3 (1:1000) were used as internal controls for loading the protein concentration.

### Immunofluorescence staining

The cryofixed sections of spinal cord were permeabilized and blocked in PBST with 1% BSA for 1 h at room temperature. The sections were then incubated overnight at 4 °C with the following primary antibodies: anti-Iba-1 (1:100), anti-Nox4 (1:100), anti-AOPP (1:100), and anti-IL-1β (1:100), anti-p-p38 (1:100), anti- p-JNK (1:100), anti- p-NF-κB p65 (1:100), anti- NLRP3 (1:100), and anti-ASC (1:100), anti- Caspase-1 (1:100), and anti-GSDMD (1:100). After rinsing with PBS, the sections were incubated for 1 h at room temperature with secondary antibodies conjugated with FITC (1:1000) or Cy5 (1:1000). The sections were then mounted with Fluoroshield containing DAPI to stain the nuclei, followed by capturing the sections with confocal microscopy.

The BV2 cells were seeded on glass coverslips in 24 well-plates. After various treatments, the cells were fixed in 4% paraformaldehyde at room temperature for 20 min and then washed thrice in PBST. After permeabilization with 0.1% Triton X-100/PBS for 15 min, the cells were washed with PBS, blocked in PBS with 5% BSA at room temperature for 1 h and then incubated with NF-κB p65 (1:100), NLRP3 (1:100), and GSDMD (1:100) primary antibodies at 4 °C overnight. After being washed with PBS, the cells were incubated with FITC-conjugated secondary antibodies (1:1000) for 1 h at room temperature. Finally, the cells were washed with PBS, mounted in Fluoroshield containing DAPI and analyzed by confocal microscopy.

### Statistical analyses

All experiments were repeated at least three times. Statistical data are presented as means ±SEM and analyzed by SPSS 20.0 (IBM, NY, USA) and GraphPad Prism 5 (GraphPad Software, CA, USA) software. One-way analysis of variance (ANOVA) was conducted with Bonferroni post hoc tests analysis to examine the differences between groups. A 푃 value of < 0.05 was considered to be statistically significant.

## Results

### AOPPs and oxidative stress levels were increased in SCI rats

**Fig. 1:**
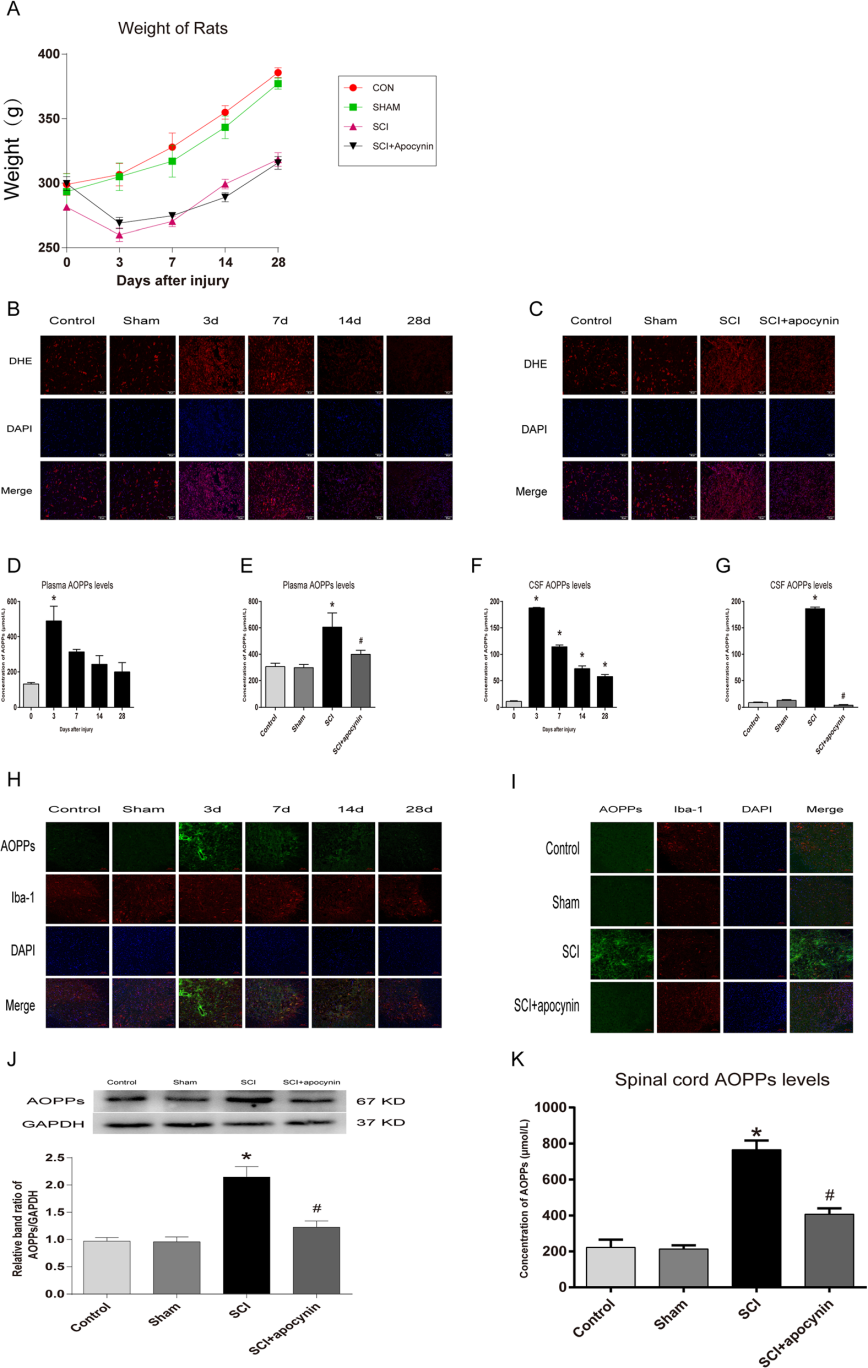
AOPPs and oxidative stress levels were increased in rats with spinal cord injury (SCI). **a** The body weight of rats was measured at different time points. **b**, **c** DHE staining of spinal cord tissues in different groups. The comparison of oxidative stress levels between SCI group and SCI apocynin group at 3 days after SCI. Scale bar, 50 μm. **d**‑**g** The concentration of AOPPs in plasma and CSF was detected by chloramine-T method at different time points and in different groups on day 3 after SCI. Data are representative of at least 3 independent experiments and the values are presented as mean ± SEM, *n* = 4 per group, *P < 0.05 versus control group. #*P* < 0.05 versus SCI group. **h**, **i** Immunofluorescence staining showed the expression of AOPPs in spinal cord tissues at different time points. The comparison of the levels of AOPPs between SCI group and SCI apocynin group was performed on day 3 after SCI. Scale bar, 100 μm. **j** Western blot analysis showed the contents of AOPPs in spinal cord in different groups on day 3 after SCI. *n* = 4 per group, *P < 0.05 versus control group. #*P* < 0.05 versus SCI group. **k** The concentration of AOPPs in spinal cord was detected by chloramine-T method in different groups on day 3 after SCI. Data are representative of at least 3 independent experiments and the values are presented as mean ± SEM, *n* = 4 per group, *P < 0.05 versus control group. #*P* < 0.05 versus SCI group

The weight of the rats after SCI was decreased all the time when compared with control rats. The body weight of rats in the SCI group and SCI + apocynin group showed no significant differences at all time points (Fig. 1a). To examine the levels of oxidative stress, DHE fluorescence staining of spinal cord was performed. Representative images showing the time course of DHE oxidation in the spinal cord after SCI (Fig. 1b). The results showed significant elevation in the levels of oxidative stress on days 3 and 7 post-injury when compared to the spinal cord of controls and sham rats. As depicted in Fig. 1c, apocynin has significantly reduced the levels of oxidative stress when compared with SCI group.

To identify whether AOPPs play an important role in SCI, the plasma, CSF, and spinal cord tissues were examined at each time point. As shown in Fig. 1 d and f, the levels of AOPPs in plasma and CSF and the contents of AOPPs in the spinal cord were significantly increased in SCI group, and reached peak on day 3 (489.848 ± 103.754 μmol/L, *P* < 0.01 in Plasma AOPPs; 187.950 ± 1.947 μmol/L, *P* < 0.01 in CSF AOPPs), followed by a gradual decrease. Apocynin significantly inhibited the levels of AOPPs in plasma and CSF (Fig. 1e, g). Immunoreactivity of anti-AOPPs was observed in the spinal cord microglia sections of the rats that are co-labeled with microglia marker, Iba-1. AOPPs were markedly increased in SCI group and apocynin partly inhibited the expression of AOPPs (Fig. 1h, i). Western blot of AOPPs also indicated that the expression of AOPPs was increased by ≈twofold after SCI, while apocynin reduced the expression by about 33% (Fig. 1j). Apocynin significantly inhibited the levels of AOPPs in spinal cord (Fig. 1k).

### Apocynin improved the recovery of forelimb motor functional and histological outcomes after SCI

To determine whether apocynin contributes to the behavioral recovery after SCI, the functional recovery of forelimbs was measured with grooming test, cylinder rearing test, and FLS.

**Fig. 2:**
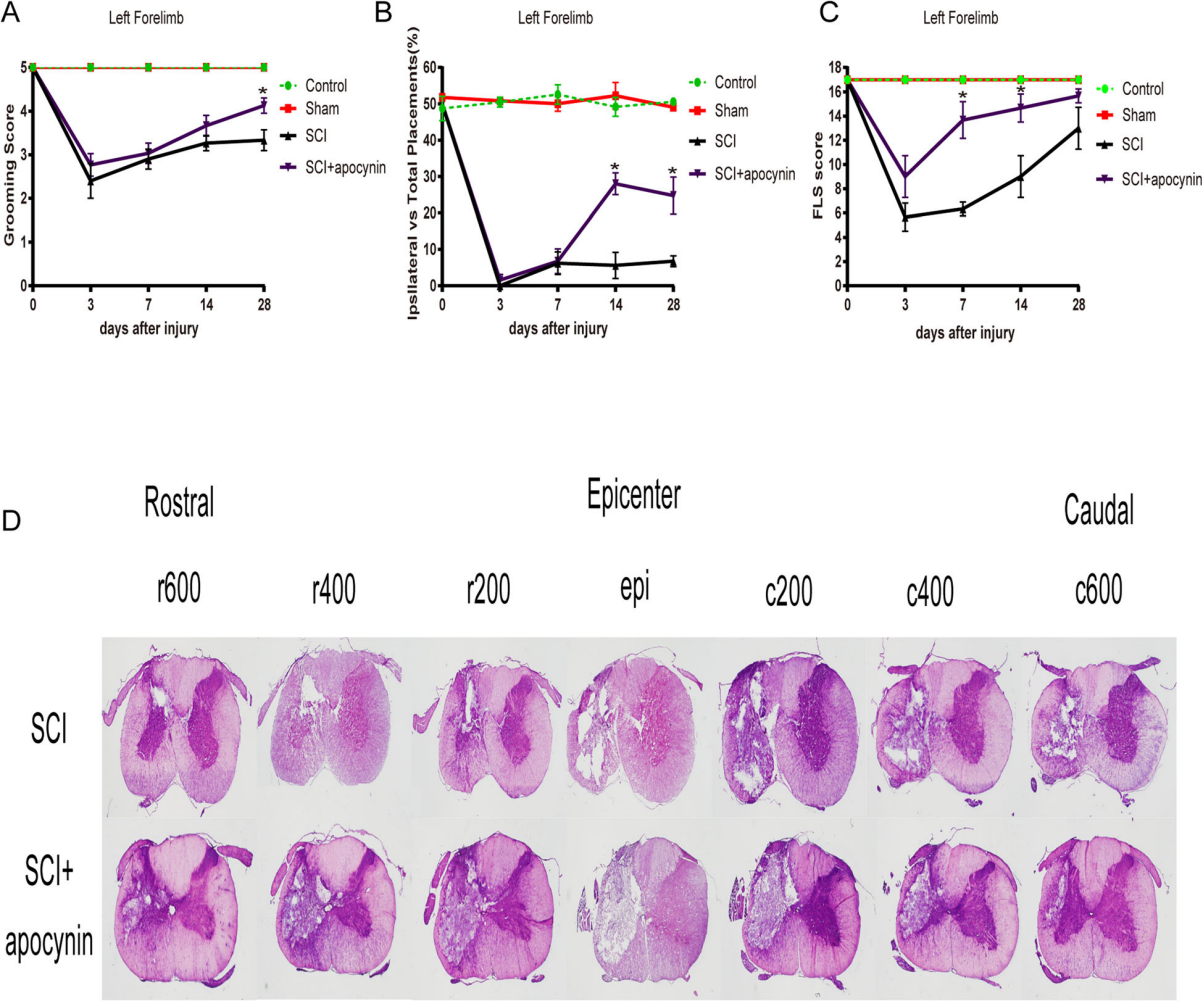
The forelimb motor functional and histological outcomes in rats after spinal cord injury (SCI). **a** The rats in SCI + apocynin group exhibited significant improvement in grooming test when compared with SCI group on day 28 after SCI. **b** The rats in SCI + apocynin group showed significant improvement in the Cylinder rearing test when compared with SCI group on days 14 and 28 after SCI. **c** The rats in SCI + apocynin group had higher scores in FLS when compared with SCI group on days 7 and 14 after SCI. **d** Photomicrographs of representative spinal cord transverse sections stained with hematoxylin-eosin at the epicenter and 200 μm increments rostral-caudal to the epicenter. On postoperative day 28, HE stained sections demonstrated reduction in the area of gray matter and lateral funiculus of white matter in SCI rats administrated with apocynin

In grooming test, all rats with ipsilateral paw after SCI showed lower grooming scores when compared to pre-injury. No significant differences were observed between SCI group and SCI + apocynin group on days 3, 7, and 14 after SCI. On day 28 after SCI, the rats in the SCI + apocynin group (4.133 ± 0.306) demonstrated a significantly higher grooming scores than those in the SCI group (3.333 ± 0.416) (Fig. 2a).

In cylinder rearing test before injury, the rats almost used both limbs equally during wall placements. After hemi-contusion, the ipsilateral paw usage was increased in SCI + apocynin group when compared with SCI group on days 14 (28.027 ± 5.203 vs 5.633 ± 6.215, respectively) and 28 (24.770 ± 8.841 vs 6.799, respectively) (Fig. 2b).

In FLS, the uninjured rats scored 17 as the preoperative baseline of FLS. Figure 2c showed that both groups demonstrated no significant differences on days 3 and 28 after SCI. Nevertheless, the SCI + apocynin group had better scores than SCI group on days 7 (13.667 ± 1.528 vs. 6.333 ± 0.577, respectively) and 14 after SCI (14.667 ± 1.155 vs. 9.000 ± 1.732, respectively), (*P* < 0.05).

To examine the protective effects of apocynin in rats with SCI more comprehensively, HE staining was performed on 20 μm frozen sections of spinal cord. The lesions were observed on the transverse sections of spinal cord from 600 μm caudal to 600 μm rostral to the epicenter. The largest lesion was observed at the epicenter. The rats in the SCI group were associated with severe damage of loss of large area of gray matter and lateral funiculus of white matter, and apocynin reduced the loss of spinal cord gray as well as white matter (Fig. 2d). These data suggested that apocynin effectively ameliorated the recovery of forelimb motor functional as well as histological outcomes in SCI rats.

### AOPPs-MSA and SCI induced microglial activation

**Fig. 3:**
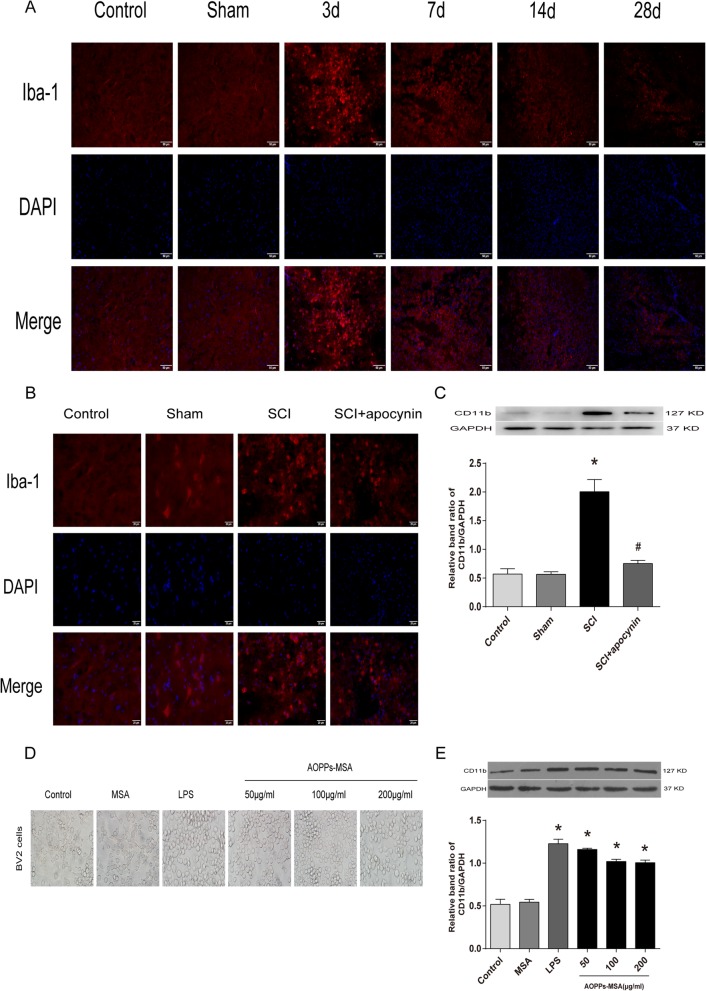
SCI and AOPPs-MSA induced microglial activation. **a** Immunofluorescence staining showed the amounts of microglia in spinal cord tissues at different time points. Scale bar, 50 μm. **b** Iba-1 staining in microglia in the ventral horn of spinal cord sections on day 3 after SCI. Activated microglia exhibited significant cellular hypertrophy and retraction of processes after SCI, while resting microglia displayed smaller somata and processes. Scale bar, 20 μm. **c** Western blot demonstrated the protein levels of CD11b in the spinal cord of different groups on day 3 after SCI. *n* = 4 per group, *P < 0.05 versus control group. #*P* < 0.05 versus SCI group. **d** BV2 cells in the control group displayed ramified shapes, while after LPS stimulation (1 μg/mL) or AOPPs, most of the cells appeared round or ameboid in shape. **e** Western blots analysis of CD11b in BV2 cells of each group. Data are representative of at least 3 independent experiments and the values are presented as mean ± SEM. *P < 0.05 versus control group

Previous study demonstrated that microglia activation is a hallmark of SCI [29]. In vivo, immunohistochemistry was conducted by using an antibody against Iba-1, and the results revealed that microglia exhibited a marked cellular hypertrophy and retraction of cytoplasmic processes after SCI, while the resting microglia in uninjured rats displayed smaller somata with processes (Fig. 3a, b). As a molecular marker of microglia, CD11b has been widely used for microglial activation. The protein levels of CD11b were increased (≈ 3.6-fold) in SCI group, while apocynin inhibited CD11b expression by about 62% (Fig. 3c). Similar to the results of in vivo experiments, the BV2 cells in the control group displayed ramified shapes, while most of the BV2 cells displayed round or ameboid shapes in vitro after LPS and AOPPs stimulation (Fig. 3d). AOPPs and LPS significantly stimulated CD11b expression in a dose-dependent manner in BV2 cells by 2.4-, 2.3-, 2.2-, and 2.2-folds over the control group (Fig. 3e). These results implied that AOPPs participated during SCI via microglial cells.

### AOPPs regulated BV2 cells to generate intracellular ROS via the activation of NADPH

**Fig. 4:**
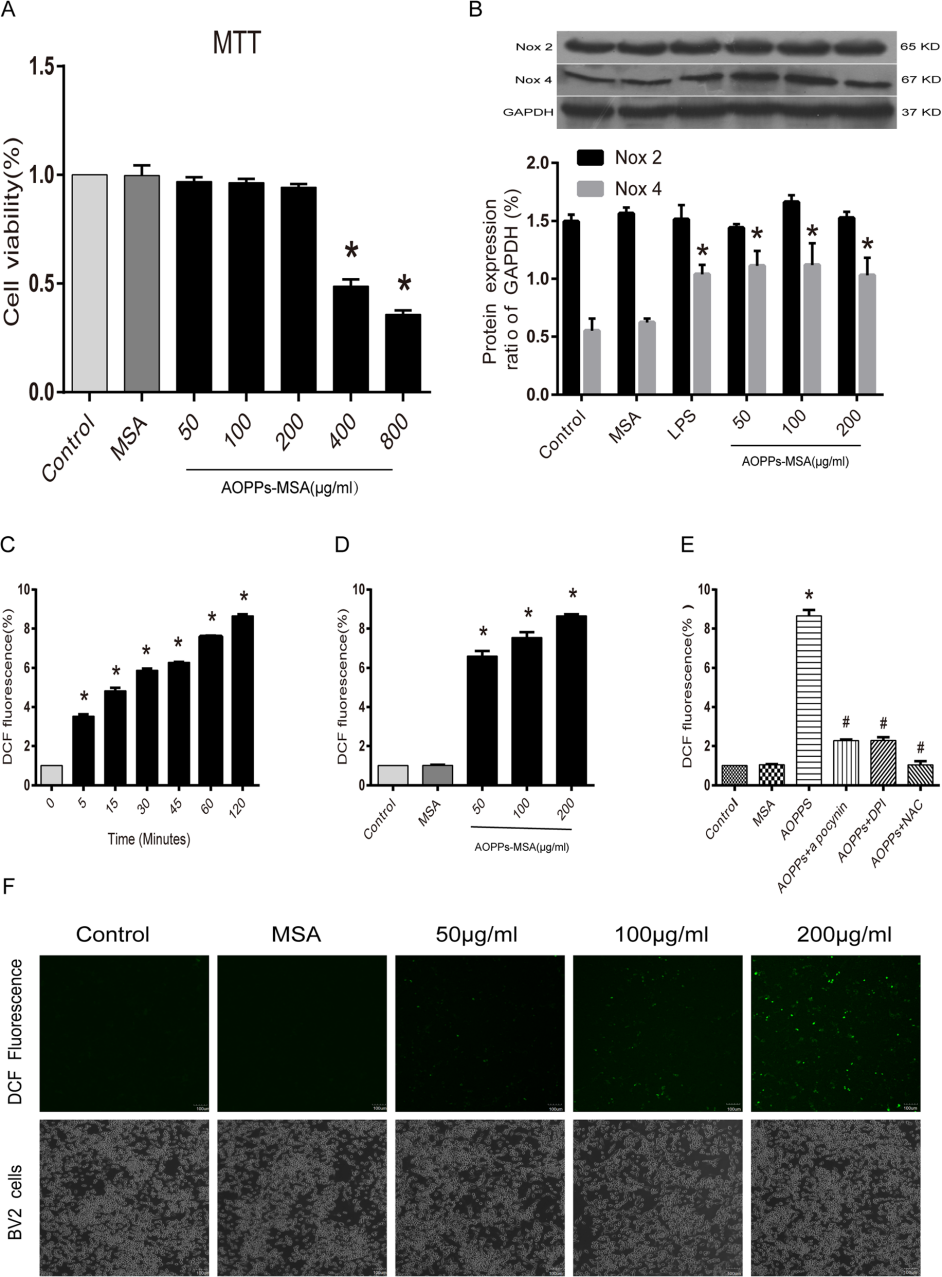
AOPPs trigger intracellular ROS production in BV2 cells via the activation of NADPH oxidase. **a** Viability of BV2 cells administrated with AOPPs-MSA in different concentrations after culturing for 24 h. **b** BV2 cells were treated with LPS (1 μg/mL) or AOPPs-MSA, and the expression levels of Nox2 and Nox4 were detected by Western blotting. Data are representative of at least 3 independent experiments and the values are presented as mean ± SEM. *P < 0.05 versus Control group. **c** BV2 cells were incubated in AOPPs-MSA (200 μg/mL) for indicated time durations prior to a 20-min DCFH-DA (10 μM) treatment. **d** BV2 cells were cultured with controls, MSA, or AOPPs-MSA for 2 h followed by a 20-min DCFH-DA (10 μM) treatment. **e** BV2 cells were treated with AOPPs-MSA (200 μg/mL) with or without apocynin (100 μM), DPI (10 μM), and NAC (5 mM) for 2 h. AOPP-induced ROS generation was significantly inhibited by NOX inhibitors (apocynin and DPI) and ROS scavenger (NAC). **f**, **e** BV2 cells were cultured with control medium, MSA, and different concentrations of AOPPs-MSA for 2 h. Confocal laser scanning microscopy was used to visualize ROS generation in BV2 cells with the use of DCFH-DA. Data are representative of at least 3 independent experiments and the values are presented as mean ± SEM. Scale bar, 100 μm. *P < 0.05 versus control (0) group. #*P* < 0.05 versus AOPPs-MSA group

Previous studies have demonstrated that microglia produced intracellular ROS via NADPH oxidase under oxidative stress conditions [30]. To verify whether AOPPs, which are oxidative stress products, could cause excessive ROS production in BV2 cells, the intracellular ROS levels in AOPPs-treated BV2 cells were examined. According to MTT test results, 400 μg/mL AOPPs-MSA significantly caused death of BV2 cells (cell viability was 48.768 ± 3.118%), while incubation in 0 to 200 μg/mL AOPPs-MSA had little effects on cell fatality (cell viability was above 95%), (Fig. 4a). Therefore, BV2 cells were cultured with increasing concentrations of AOPPs-MSA (0, 50, 100, and 200 μg/mL) at different time points (0, 5, 15, 30, 45, 60, and 120 min). The expression level of Nox4 was increased after stimulation, while the protein levels of Nox2 showed no significant differences by Western blotting (Fig. 4b). ROS production was increased in BV2 cells cultured with AOPPs-MSA in a dose- and time-dependent manner (Fig. 4c, d). The increased ROS production was further confirmed by confocal microscopy (Fig. 4f).

To determine the role of NADPH oxidases in ROS generation, BV2 cells were pretreated with ROS scavenger N-acetylcysteine (NAC), NOX inhibitors diphenylene iodonium (DPI) and apocynin. As shown in Fig. 4e, the AOPP-induced ROS generation was significantly inhibited in BV2 cells pretreated with NAC, DPI, or apocynin separately (*P* < 0.05).

### SCI increased the expression of Nox4, p-p38,p-JNK, p-NF-κB p65, Caspase-1 P10, N-GSDMD, TNF-α, and IL-1β in vivo

**Fig. 5:**
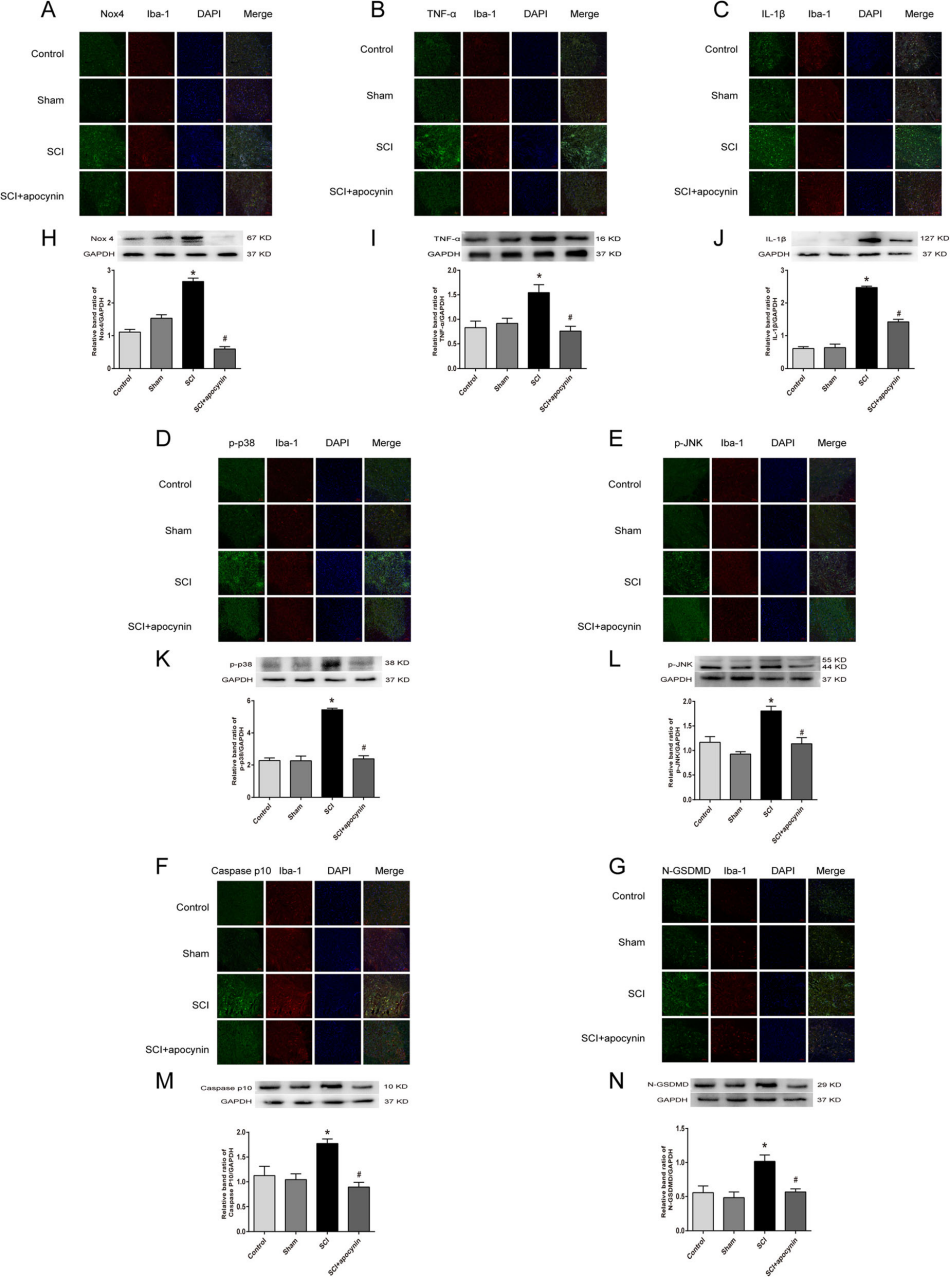
Expression levels of Nox4, TNF-α, IL-1β, p-p38, p-JNK, Caspase p10, and N-GSDMD were detected in the spinal cord of rats on day 3 after SCI. **a**‑**g** Immunofluorescence staining of spinal cord sections in each group. **h**‑**n** The expression levels of the above proteins were analyzed by Western blotting. Data are representative of at least 3 independent experiments and the values are presented as mean ± SEM. Scale bar, 100 μm *n* = 4 per group. *P < 0.05 versus control group. #*P* < 0.05 versus SCI group

Spinal cords were used for immunofluorescent staining and western blotting analysis on day 3 after SCI. The results of immunofluorescent staining revealed that the expressions of Nox4, p-p38, p-JNK, p-NF-κB p65, Caspase-1 P10, N-GSDMD, TNF-α, and IL-1β were markedly increased in SCI group when compared with control group (Fig. 5a‑g). To strengthen our results, western blot analysis was performed to evaluate the expression levels of Nox4, p-p38, p-JNK, p-NF-κB p65, Caspase-1 P10, N-GSDMD, TNF-α, and IL-1β. Figure 5h‑n revealed that the protein levels of Nox4, p-p38, p-JNK, p-NF-κB p65, Caspase-1 P10, N-GSDMD, TNF-α, and IL-1β were significantly increased in SCI group. Meanwhile, the rats after apocynin treatment showed efficient reduction in the expression of the above proteins.

### AOPPs induced inflammation in BV2 cells through Nox4-ROS-MAPK- NF-κB pathway

**Fig. 6:**
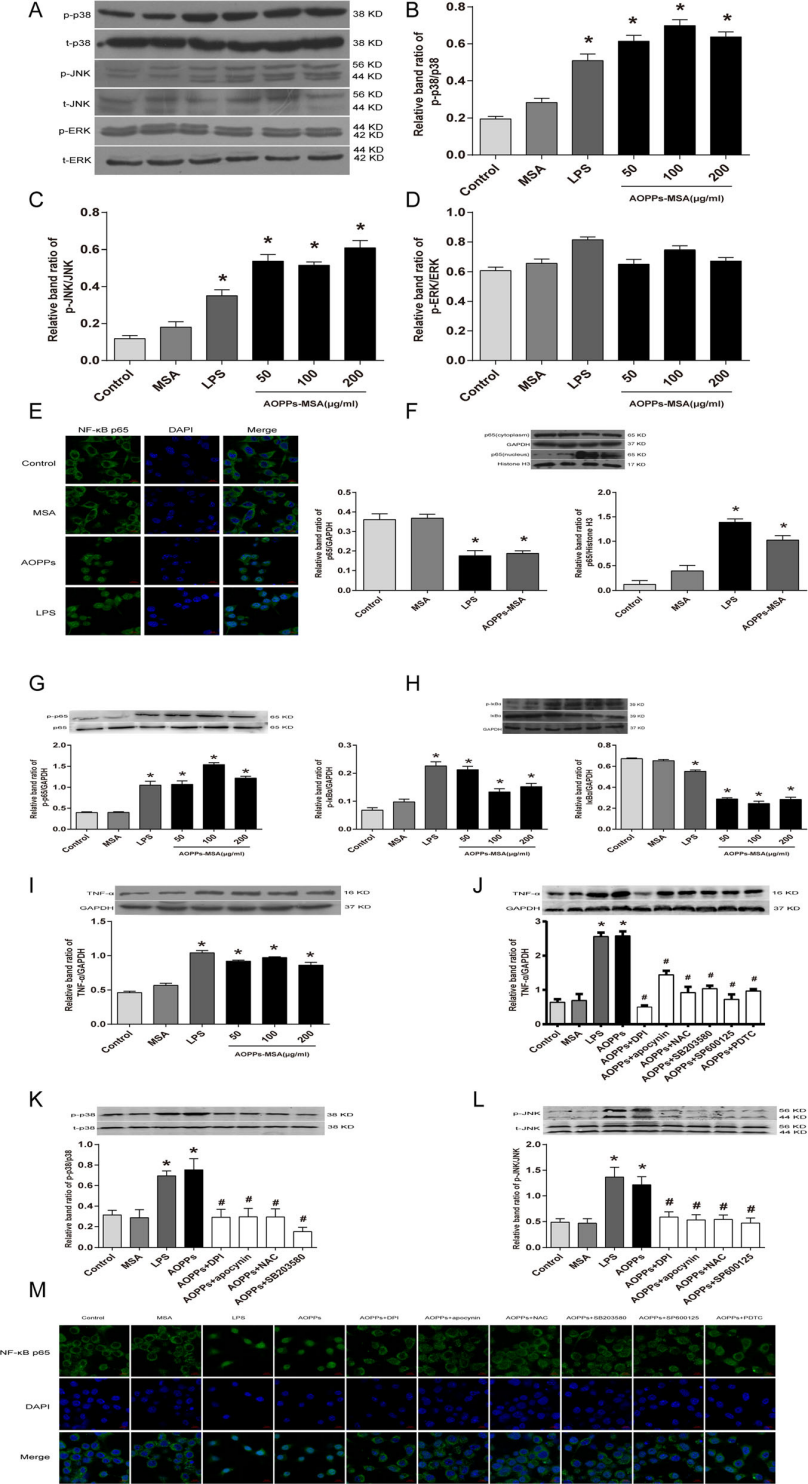
AOPPs induced inflammation in BV2 cells through Nox4-ROS-MAPKs-NF-κB signaling pathway. **a**‑**d** Effects of AOPPs-MSA on p-p38, p-JNK, and p-ERK at different concentrations after incubation for 24 h. The levels of p-p38, p-JNK, and p-ERK were quantified and normalized with their respective total p38, JNK, or ERK levels. Data are representative of at least 3 independent experiments and the values are presented as mean ± SEM. *P < 0.05 versus control group. **e** Nuclear translocation of p65/NF-κB subunit was carried out by immunocytochemistry method. BV2 cells were labeled with anti-p65 antibodies (red) and DAPI (blue). Representative images were obtained from three independent experiments. Scale bar, 10 μm. **f**‑**h** The expression levels of p-IκB-α, IκB-α, p- p65, p65, and NF-κB p65 in nuclear or cytoplasm were measured by western blotting analysis. GAPDH (cytoplasm) and histone H3 (nuclear) were used as internal controls. **i** The levels of TNF-α were significantly increased in BV2 cells incubated with AOPPs-MSA. **j**‑**l** BV2 cells were pre-incubated with apocynin (100 μM), DPI (10 μM), NAC (5 mM), a p38 inhibitor (SB203580, 10 μM), a JNK inhibitor (SP600125, 100 μM), and a specific NF-κB inhibitor (PDTC, 100 μM) before AOPPs-MSA (200 μg/mL) treatment. The protein levels of TNF-α, p-p38, p-JNK, and GAPDH were measured by western blot analysis. (M) NF-κB p65 antibody conjugated with FITC, and nuclei was labeled with DAPI. Scale bar, 10 μm. Data are representative of at least 3 independent experiments and the values are presented as mean ± SEM. *P < 0.05 versus control group. #*P* < 0.05 versus AOPPs-MSA group

Activation of MAPKs has long been thought to play a pivotal role in inflammatory response by modulating the expression of pro-inflammatory cytokines in microglia [31]. Western blotting showed that treatment with LPS and AOPPs-MSA (50,100, and 200 μg/mL) dramatically and rapidly increased the phosphorylation of p38 and JNK, but did not influence the phosphorylation levels of ERK (Fig. 6a‑d).

Since NF-κB is an essential and ubiquitous transcription factor for expressing various pro-inflammatory mediators such as TNF-α, IL-1β, COX2, and iNOS in microglia cells [32], we thus determined whether AOPPs affected NF-κB activation in the microglia by immunofluorescence analysis and western blotting. Confocal microscopy system showed that the NF-κB p65 subunit was primarily located in the cytosol in untreated conditions (Fig. 6e). When BV2 cells were exposed to LPS and AOPPs (200 μg/mL) for 24 h, the p65 protein appeared both in the cytoplasm as well as in the nucleus. To further verify the induction of nuclear translocation of NF-κB p65 by AOPPs, western blotting was performed to confirm the induction of NF-κB p65 subunit translocation by AOPPs into the nucleus (Fig. 6f, g). As IκB-α is known to be an inhibitory subunit of NF-κB complex that prevents nuclear translocation of NF-κB, the expression levels of IκB-α and phospho- IκB-α proteins were analyzed by western blotting. As expected, the AOPPs significantly induced IκB-α degradation and increased IκB-α phosphorylation (Fig. 6h).

To clarify whether Nox4-ROS-MAPK- NF-κB signaling pathway participated in AOPPs induced inflammation, BV2 cells were stimulated with increasing concentrations of AOPPs-MSA (50, 100, and 200 μg/mL) for 24 h. Figure 6i showed that the expression of TNF-α was rapidly increased. The characteristics of Nox4-ROS-MAPK signaling pathway was investigated by Western blotting. BV2 cells were pretreated with DPI, apo, and NAC. The results showed that the expression of p-p38 and p-JNK were significantly decreased (Fig. 6k, l). Meanwhile, immunocytochemistry analysis and western blotting were performed to confirm the critical role of Nox4-ROS-MAPK-NF-κB during inflammatory response of BV2 cells. DPI, apo, NAC SB203580 (a p38 inhibitor), SP600125 (a JNK inhibitor), and PDTC (a NF-κB inhibitor) were used for culturing with BV2 cells. Confocal microscopy showed that the nuclear translocation of NF-κB p65 was significantly inhibited under these conditions (Fig. 6m). The protein levels of TNF-α were then obviously antagonized after treatment with the above inhibitors (Fig. 6j). Together, our results provided the evidence that Nox4-ROS-MAPK-NF-κB signaling pathway participated in inflammatory response mediated by AOPPs in BV2 cells.

### AOPPs induced pyroptosis in BV2 cells

**Fig. 7:**
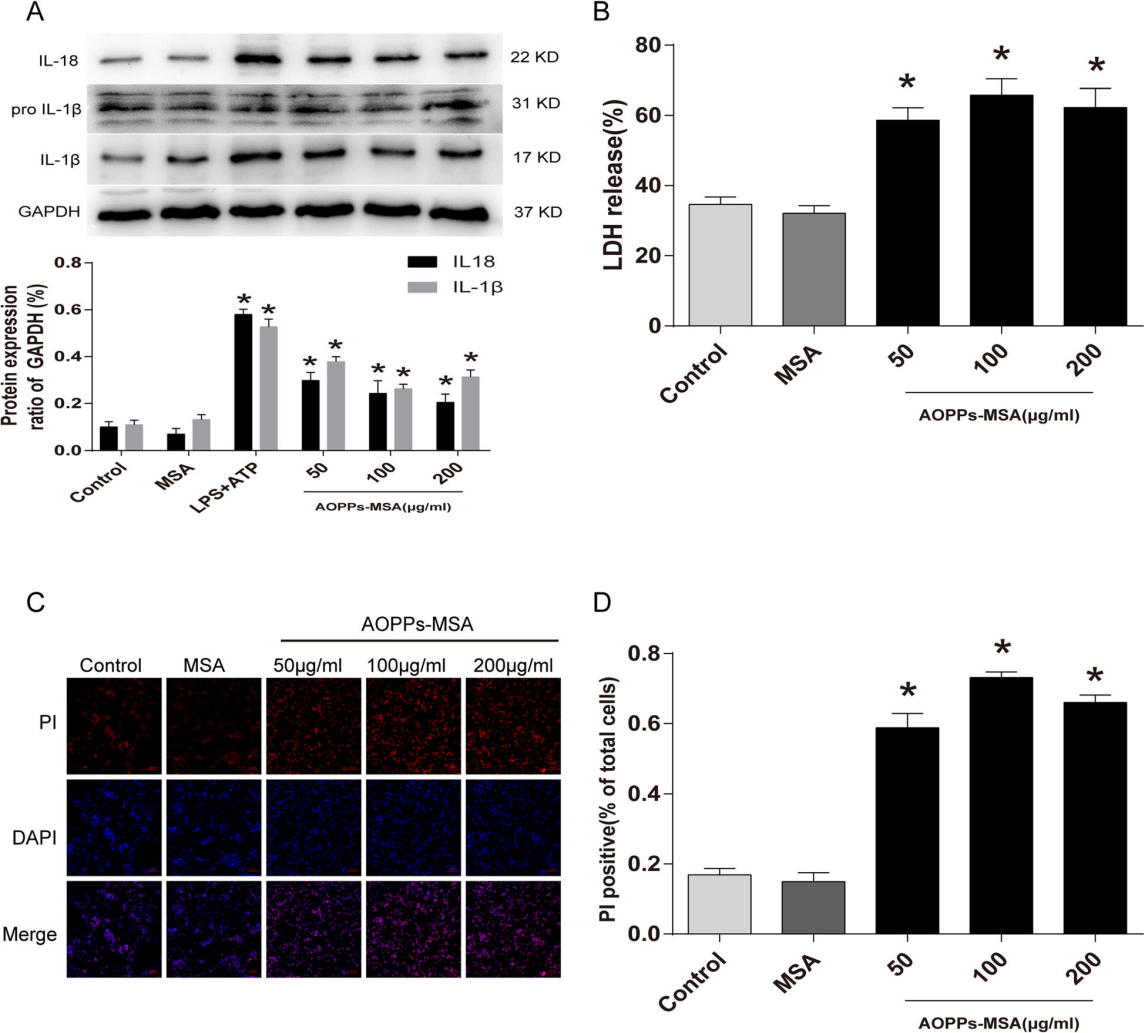
Effects of AOPPs-MSA on pyroptosis in BV2 cells. **a** BV2 cells were administered with LPS (1 μg/mL), ATP (5 mM), and different concentrations of AOPPs-MSA. Western blotting analysis was performed to determine the expression of IL-18, pro IL-1β, and IL-1β. Data are representative of at least 3 independent experiments and the values are presented as mean ± SEM. *P < 0.05 versus control group. **b** Effect of different concentrations of AOPPs-MSA induced LDH release. **c** BV2 cells were double stained by PI (red) and DAPI (blue) and pictures were captured by using confocal microscopy. **d** PI positive cells were calculated in five random fields. Data are representative of at least 3 independent experiments and the values are presented as mean ± SEM. Scale bar, 100 μm. *P < 0.05 versus control group

Two markers of pyroptosis were detected to elucidate whether AOPPs could trigger pyroptosis in BV2 cells, LDH release, and PI uptake. As described in Fig. 7b, AOPPs and LPS after treatment with ATP significantly increased the levels of LDH release (1.7-, 1.9-, and 1.8-fold increases). Moreover, similar results were obtained by PI staining, in which the incubated cells with AOPPs exhibited a significantly higher ratio of PI positive cells (58.856 ± 8.087%, 73.155 ± 3.237%, and 66.004 ± 4.266%) than the untreated cells (Fig. 7c, d). Additionally, the protein levels of mature IL-1β and IL-18 were significantly upregulated by treatment with AOPPs (Fig. 7d).

### AOPPs triggered the activation of NLRP3 inflammasome

**Fig. 8:**
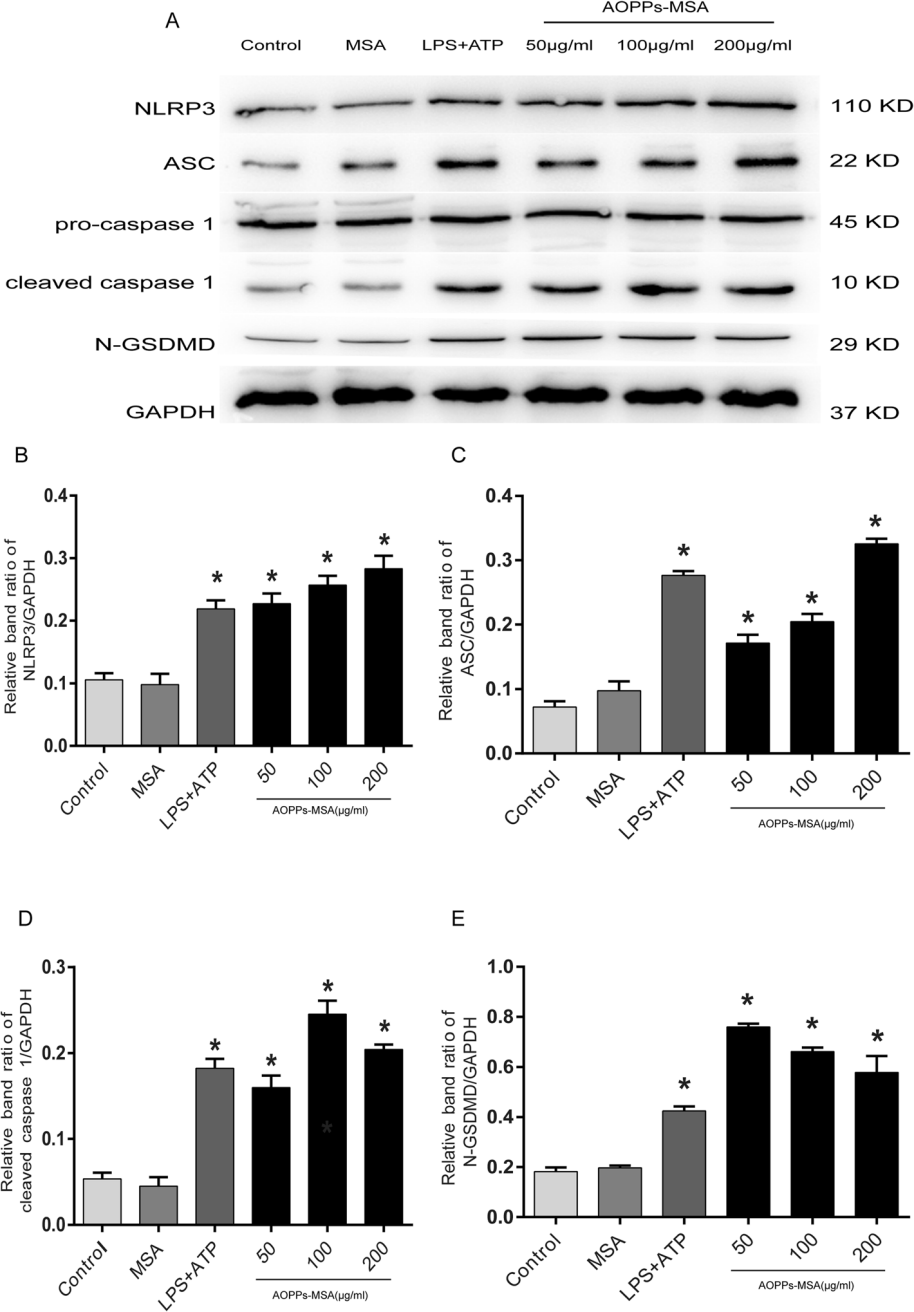
Effects of AOPPs-MSA on NLRP3 inflammasome in BV2 cells. **a** BV2 cells were incubated with AOPPs-MSA (24 h) and 1 μg/mL LPS (24 h) with 5 mM ATP (1 h). **b**‑**e** Western blotting was performed to determine the expression of NLRP3, ASC, Caspase p10, N-GSDMD, and GAPDH. Data are representative of at least 3 independent experiments and the values are presented as mean ± SEM. *P < 0.05 versus control group

Studies suggested that the activation of NLRP3 inflammasomes triggered pyroptosis, promoting the secretion of IL-1β and IL-18. Moreover, activation of caspase-1 remains a critical step for inflammasomes to mediate pyroptosis and IL-1β/IL-18 production. GSDMD is a substrate of caspase-1 and the N-terminal proteolytic fragment of GSDMD triggers cell pyroptosis and IL-1β secretion [33]. Therefore, western blot analysis was performed to verify the relative proteins. As demonstrated in Fig. 8a‑e, BV2 cells were cultured with LPS + ATP and AOPPs-MSA (50,100, and 200 μg/mL), and the results showed that the expression of NLRP3, ASC, Caspase-1 p10, and N-GSDMD was significantly increased.

### AOPPs induced pyroptosis in BV2 cells through Nox4-ROS-NLRP3-GSDMD signaling pathway

**Fig. 9:**
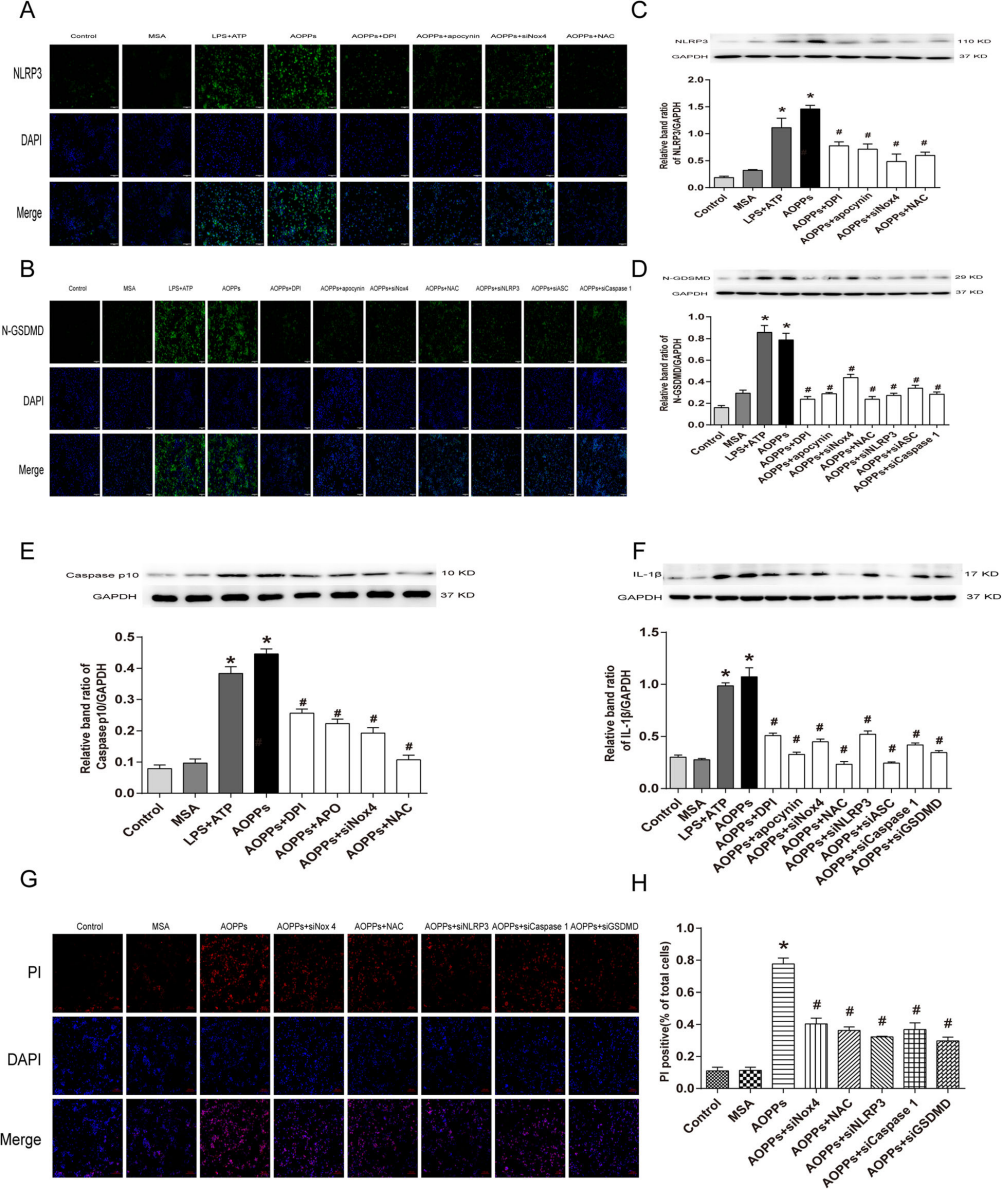
AOPPs induced pyroptosis in BV2 cells through Nox4-ROS-NLRP3-GSDMD signaling pathway. **a**, **b** Confocal microscopy was used to observe the expression changes in different groups. NLRP3 and N-GSDMD antibody conjugated with FITC, and nuclei was labeled with DAPI. Scale bar, 100 μm. **c**‑**f** BV2 cells were pre-incubated with apocynin (100 μM), DPI (10 μM), NAC (5 mM), siNox4, siNLRP3, siASC, siCaspase 1, and siGSDMD before AOPPs-MSA (200 μg/mL) treatment. The protein levels of NLRP3, N-GSDMD, Caspase p10, N-GSDMD, and GAPDH were normalized by western blotting analysis. Data are representative of at least 3 independent experiments and the values are presented as mean ± SEM. *P < 0.05 versus control group. #*P* < 0.05 versus AOPPs-MSA group. **g**, **h** Double staining of PI (red) and DAPI (blue). PI positive cells were significantly reduced after pre-treatment with NAC (5 mM), siNox4, siNLRP3, siCaspase 1, and siGSDMD. Data are representative of at least 3 independent experiments and the values are presented as mean ± SEM. Scale bar, 100 μm. *P < 0.05 versus control group. #*P* < 0.05 versus AOPPs-MSA group

To further elucidate that pyroptosis of BV2 cells induced by AOPPs was mediated by Nox4-ROS-NLRP3 signaling pathway, BV2 cells were pretreated with DPI, apo, NAC, transfected with siNLRP3, siASC, siCaspase-1 and siGSDMD, and then stimulated with AOPPs (200 μg/mL). As described in Fig. 9c‑f, the proteins level of NLRP3, N-GSDMD, Caspase-1 p10, and IL-1β were markedly decreased under these conditions. To further confirm these results, immunocytochemistry analysis of NLRP3 and Caspase-1 p10 were performed. Immunofluorescent staining results revealed that the expression of NLRP3 and Caspase-1 p10 was obviously decreased when compared with AOPPs group (Fig. 9a, b). Meanwhile, PI staining showed that these treatments significantly decreased the PI uptake levels when compared with AOPPs group (*P* < 0.05) (Fig. 9g, h). These results indicated that AOPPs triggered pyroptosis in BV2 cells mediated by Nox4-ROS-NLRP3-GSDMD signaling pathway.

## Discussion

The present study identified the increasing levels as well as contents of AOPPs in a rat SCI model. In vitro evidences were provided to confirm that AOPPs induced inflammation in BV2 cells via ROS-dependent involving MAPK-NF-κB signaling pathway and NLRP3-GSDMD. In in vivo study, NADPH oxidase inhibitor apocynin was used to inhibit oxidative stress induced by AOPPs in SCI rats. To the best of our knowledge, this is the first report to demonstrate the pathogenic effect of AOPPs in SCI.

Secondary injury occurs within minutes to weeks following primary damage and is accompanied with inflammatory response that is characterized by neutrophil infiltration and microglial activation, which leads to the release of ROS and pro-inflammatory cytokines [3, 34]. Meanwhile, secondary injury induced inflammatory response plays a crucial role in the pathogenesis after SCI. AOPPs were first discovered and defined by Witko-Sarsat in the plasma of uremic patients receiving maintenance dialysis [20]. Several studies have suggested that the formation and accumulation of plasma AOPPs served as mediators of inflammation and took part in a variety of diseases [18, 27]. Recent studies have showed the accumulation of plasma AOPPs in different neuroinflammatory disorders [19, 35], whereas little is known about their pathogenic role and mechanism in SCI.

In the current study, a significant increase in the intensity of DHE staining was observed, which was in line with the consensus that oxidative stress has been considered as a hallmark of SCI. Meanwhile, proteins in plasma, CSF, and spinal cord are easily oxidized by chlorinated oxidants to form AOPPs after SCI. In this study, the AOPPs of plasma and CSF and the content of AOPPs in the spinal cord of SCI rats were significantly increased when compared with uninjured rats. This is the first evidence for the accumulation of AOPPs in rat SCI model. These results indicated that AOPPs might participate in the pathological process of SCI, highlighting the urgent need to understand their effects on cells, tissues, and organs under physiological conditions and explore their underlying mechanisms. For in vivo experiments, we collected the blood, cerebrospinal fluid and spinal cord, from rats in the study, and directly measured the actual concentration of AOPPs, rather than the protein concentration. Hence, the unit of AOPPs in the blood, cerebrospinal fluid, and spinal cord was μmol/L. AOPPs-MSA was prepared by HOCL and mouse serum albumin (MSA), in vitro. The configured AOPPs-MSA contains a certain amount of AOPPs, but not all AOPPs-MSA are AOPPs. According to our studies, 1 g/L AOPPs-MSA contains about 50.50 ± 2.80 μmol/L AOPPs. Thus, 200 μg/mL AOPPs-MSA contains about 10.1 ± 0.56 μmol/L AOPPs. So, the concentration of AOPPs in vitro was under the concentrations of AOPPs measured in the plasma, cerebrospinal fluid, and spinal cord after spinal cord injury.

In in vivo experiments, the rats were administered with NADPH oxidase inhibitor apocynin after SCI. The results showed that SCI rats after apocynin treatment displayed a better recovery of forelimb motor function when compared with SCI rats. Corresponding to the behavioral assessments, HE staining also showed improvement in histological outcomes in the apocynin group. These results demonstrated that the blockage of oxidative stress by apocynin could alleviate secondary damage after SCI.

Previous literature suggested that SCI is associated with inflammatory response that is characterized by microglial activation [36]. We then hypothesized the relationship between AOPPs and microglia after SCI. BV2 cells were activated by AOPPs resulting in morphological change. Similar to BV2 cells, microglial cells in the spinal cord were activated after SCI. Meanwhile, apocynin inhibited the activation of microglia cells. CD11b, a molecular marker of microglial activation, showed robust upregulation both in in vivo and in vitro. These data showed that AOPPs participated in the pathological progression of SCI by targeting microglia in the spinal cord.

As reported previously, emerging studies have pointed out ROS production as a key mediator for modulating intracellular signaling activation in a variety of pathologies. Also, AOPPs significantly increased ROS production at different concentrations and at different time points. According to previous reports, NADPH oxidase is regarded as one of the main sources of ROS production that plays a pivotal role as a modulator in ROS-mediated oxidative damage in many kinds of cells. It has been well demonstrated that AOPPs could induce ROS generation by sensitizing NADPH oxidase [37–39]. In general, 5 isoforms of NADPH oxidase (NOX) family have been identified (NOX1, 2, 3, and 4, and DUOX 2), and are shown to express in the spinal cord. Microglia are reported to express only NOX2 and NOX4 in the spinal cord and are upregulated after traumatic CNS injury [40, 41]. NOX2 is present in all cell types, NOX3 is found only in neurons, and NOX4 is found only in acute glial cells [42]. Our results showed that AOPPs could dramatically upregulate NOX4. Interestingly, there was a little impact on the regulation of NOX2. NOX inhibitors (apo, DPI) and ROS scavenger (NAC) demonstrated effective attenuation of AOPPs-induced ROS production. NOX4 expression in the spinal cord after SCI was also increased and inhibited by apocynin. These findings indicated that NADPH oxidase-mediated ROS generation as the key factor to promote AOPP-induced neuroinflammation.

MAPK family (JNK, p38, and ERK) and NF-κB are the key signaling molecules that are involved in the production of cytokines and mediators associated with inflammatory response [43]. To evaluate whether MAPK and NF-κB took part in the inflammatory response in microglia, BV2 cells were pretreated with AOPPs for 24 h. The results showed that AOPPs could induce phosphorylation of p38 and JNK, whereas little effect was observed on ERK. Furthermore, the AOPPs induced IκB-α phosphorylation, degradation, and nuclear translocation of p65 in BV2 cells. Excessive ROS generation induced inflammatory signaling cascades in microglia through activation of MAPK, which in turn led to the activation of NF-κB and expression of a variety of pro-inflammatory cytokines, such as TNF-α, IL-1β, and iNOS. In accordance with in vitro experiments, the expression of p-p38, p-JNK, and p-p65 was increased in SCI rats, whereas decreased in SCI rats treated with apocynin. In consistent with these findings, a series of inhibitors such as apo, DPI, NAC, SB203580, SP600125, and PDTC were used. The results showed that blocking NOX4-mediated ROS generation suppressed the phosphorylation of p38 and JNK. Next, suppression of NOX4, ROS, and MAPK activities reduced AOPPs induced NF-휅B activation. Finally, IL-1β and CD11b were significantly decreased by inhibiting Nox4, ROS, MAPK, and NF-κB activities. Taken together, these results suggested that AOPPs might act as key mediators for initiating Nox4-ROS-MAPK- NF-κB signaling pathway.

Pyroptosis is characterized by pore formation in the plasma membrane, cell swelling, and rupture of the membrane, and played a critical role in several CNS diseases. Recent studies suggested that pyroptosis plays a critical role in IL-18 and IL-1β release [44–46]. Therefore, pyroptosis was assessed by LDH release, PI staining, and protein levels of IL-18 and IL-1β. Our data showed that IL-18 and IL-1β were increased significantly in BV2 cells treated with AOPPs. LDH release and PI uptake were rapidly increased by treatment with AOPPs. Activation of NLRP3 inflammasome remains a crucial step to mediate cleavage of GSDMD, pyroptosis, and IL-1β/IL-18 production in the microglia. NLRP3 inflammasome has been investigated in several CNS diseases, such as traumatic spinal cord injury and Alzheimer’s disease [13, 47]. Among the members of gasdermin family, GSDMD participates in microglial pyroptosis. The cleavage of gsdmd-N domain promotes cell membrane breakage, ultimately leading to pyroptosis. In accordance with the above views, NLRP3, ASC, Caspase p10, and N-GSDMD showed significant increase after treatment with AOPPs. Also, SCI rats showed significantly high expression levels of Caspase-1 P10 and N-GSDMD than uninjured rats. Meanwhile, apocynin could reduce the expression of Caspase-1 P10 and N-GSDMD in SCI rats.

There are considerable reports that demonstrated NADPH oxidase-derived ROS as a triggering factor to activate NLRP3 inflammasome in pathological diseases [48–50]. Meanwhile, GSDMD is a substrate of caspase-1, and the N-terminal fragment of GSDMD generated by caspase-1 cleavage is responsible for executing pyroptosis and promoting the secretion of matured IL-1β and IL-18 [51, 52]. To evaluate the consistency of these results with the previous reports, treatment with inhibitors (apo, DPI, NAC) and siRNA (siNOX4, siNLRP3, siASC, siCaspase-1, siGSDMD) experiments were carried out. These results showed that the activation of NLRP3 was inhibited after blocking NOX4-derived ROS generation. Furthermore, suppression of NOX4-derived ROS and the activation of Caspase 1 blocked the cleavage of N-GSDMD. This in turn reduced the maturation and release of IL-1β by blocking NOX4-derived ROS, NLRP3 inflammasome activation, and GSDMD cleaved activity. These data demonstrated that AOPPs possibly induced IL-1β via Nox4-ROS-NLRP3- GSDMD cascade pathway.

## Conclusion

In conclusion, our findings outlined the important role of AOPPs in SCI rats. A novel mechanism of induction of AOPPs of NOX4-derived ROS to activate MAPKs, NF-κB, and NLRP3 inflammasome in the microglia after SCI was identified. Hence, this pathway might provide a new approach in preventing and mitigating the pathogenesis of SCI.

## Data Availability

All data supporting the conclusions of this manuscript are provided in the text and figures.

## Abbreviations

AOPPs: Advanced oxidation protein products
BBB: Basso, Beattie, and Bresnahan
BSA: Bovine serum albumin
CNS: Central nervous system
CSF: Cerebrospinal fluid
DMSO: Dimethyl sulfoxide
DPI: Diphenylene iodonium
apo: Apocynin
ERK: Extracellular signal-regulated kinase
FLS: Forelimb locomotor scale
GSDMD: Gasdermin-d
H&E: Hematoxylin and eosin
LDH: Lactate dehydrogenase
MAPKs: Mitogen-activated protein kinases
MTT: Thiazolyl blue terazolium bromide
NAC: N-acetylcysteine
NF-κB: Nuclear factor-κB
NOX: NADPH oxidase
PBS: Phosphate-buffered saline
ROS: Reactive oxygen species
SCI: Spinal cord injury
siRNA: Small interfering RNA
